# MRI monitoring of macaque monkeys in neuroscience: Case studies, resource and normative data comparisons

**DOI:** 10.1016/j.neuroimage.2021.117778

**Published:** 2021-04-15

**Authors:** Fabien Balezeau, Jennifer Nacef, Yukiko Kikuchi, Felix Schneider, Francesca Rocchi, Ross S. Muers, Rocio Fernandez-Palacios O'Connor, Christoph Blau, Benjamin Wilson, Richard C. Saunders, Matthew Howard, Alexander Thiele, Timothy D. Griffiths, Christopher I. Petkov, Kathy Murphy

**Affiliations:** a Biosciences Institute, Newcastle University, Newcastle upon Tyne, United Kingdom; b Comparative Biology Centre, Newcastle University, Newcastle upon Tyne, United Kingdom; c Laboratory of Neuropsychology, National Institutes of Health (NIMH), Bethesda, MD, United States; d Department of Neurosurgery, University of Iowa, Iowa City, IA, United States

**Keywords:** Magnetic resonance imaging, Primate, Welfare, Neurology, Monitoring, Diagnosis, Treatment, Resource, PRIME-DE

## Abstract

Information from Magnetic Resonance Imaging (MRI) is useful for diagnosis and treatment management of human neurological patients. MRI monitoring might also prove useful for non-human animals involved in neuroscience research provided that MRI is available and feasible and that there are no MRI contra-indications precluding scanning. However, MRI monitoring is not established in macaques and a resource is urgently needed that could grow with scientific community contributions. Here we show the utility and potential benefits of MRI-based monitoring in a few diverse cases with macaque monkeys. We also establish a *PRIMatE MRI Monitoring* (PRIME-MRM) resource within the PRIMatE Data Exchange (PRIME-DE) and quantitatively compare the cases to normative information drawn from MRI data from typical macaques in PRIME-DE. In the cases, the monkeys presented with no or mild/moderate clinical signs, were well otherwise and MRI scanning did not present a significant increase in welfare impact. Therefore, they were identified as suitable candidates for clinical investigation, MRI-based monitoring and treatment. For each case, we show MRI quantification of internal controls in relation to treatment steps and comparisons with normative data in typical monkeys drawn from PRIME-DE. We found that MRI assists in precise and early diagnosis of cerebral events and can be useful for visualising, treating and quantifying treatment response. The scientific community could now grow the PRIME-MRM resource with other cases and larger samples to further assess and increase the evidence base on the benefits of MRI monitoring of primates, complementing the animals’ clinical monitoring and treatment regime.

## Introduction

Magnetic Resonance Imaging (MRI) is available in many hospitals and institutions and is established as an important tool for the diagnosis and monitoring of treatment responses for a host of neurological events or whenever complications arise. For human clinical patients the literature provides a substantial amount of information on the types of scans that can be used, how they can be interpreted to inform diagnosis and how information in cases can be compared with normative data or with data from larger patient samples (Bradley Jr, 1993). However, outside of studies using MRI to evaluate the effects of disease models (e.g., Parkinson's disease (Luan et al., 2008), stroke (Li et al., 2018)), the same type of approach is not always available or used in work with research animals. Rarely are cases reported, and even when MRI data is available (Sadoun et al., 2015; Doane et al., 2018) few groups share them (Bridge et al., 2019). There is a pressing need for MRI monitoring information in nonhuman primates as part of the animals’ general veterinary clinical monitoring and treatment. Also, given the rarity of such cases and how few any one institution might have, it is important to establish a resource to which the global scientific community can contribute, such as within the PRIMatE Data Exchange, PRIME-DE (Milham et al., 2018, 2020). This will help to advance the 3Rs and animal welfare (Prescott and Poirier, 2020) and support delivery on scientific and biomedical advances (see Editorial Introduction this issue; Basso et al., 2021).

Non-human primates (NHP) remain indispensable animal models for understanding the human brain and for advancing preclinical and clinical treatment options for a host of brain disorders (Mitchell et al., 2018; Lemon, 2018; Roelfsema and Treue, 2014). MRI as used with human neurology patients could help to catch unexpected events early, inform diagnosis and help to monitor clinical treatment response. Refinements to neuroscientific and neurosurgical procedures with research animals have meant that complications are rare (Pickard et al., 2013), but even if and when they arise prompt diagnosis and treatment are crucial, including being able to monitor treatment response, in order to minimise suffering and to ensure the validity of scientific information.

Historically diagnostic tools for research animals have been limited, with a necessary reliance on clinical signs being both observable and relatively overt. However, this can potentially lead to late detection of disease or complications, which decreases the likelihood that treatment will be as effective or lead to full resolution. Moreover, without information about the underlying cause of the clinical signs, choosing the most appropriate treatment at the most appropriate time is extremely challenging without additional diagnostic information. In some cases, animals may have to be euthanised before a diagnosis can be reached because of uncertainty on the basis for their condition or prognosis. Furthermore, by the time symptomatic treatments are given, their condition may deteriorate and the efficacy of the treatment depend on clear indicators of improvement, such as what can be observed overtly in the animal's behaviour.

We report on the utility of MRI-based monitoring for macaques monkeys involved in scientifically important neuroimaging or neurophysiological study, establish a resource within PRIME-DE for *PRIMatE MRI Monitoring* (PRIME-MRM) and draw information from typical monkeys in PRIME-DE as normative data, to which the reported cases are quantitatively compared. In all four of the cases the monkeys presented with none or only subtle to mild/moderate symptoms, were otherwise healthy and able to interact socially, to feed and forage. Hence they were not candidates for euthanasia at the time of presentation and were identified as candidates for MRI-based diagnosis, monitoring and treatment. MRI monitoring led to information critically important for the diagnosis and treatment management of all of the cases. Thereby, MRI monitoring, if feasible, could be an invaluable addition to clinical management as is the case with human neurology patients. With the general availability of MRI scanners and the growth of primate MRI scanning data and information sharing (Milham et al., 2018, 2020), we encourage the primate neuroscience community, whenever possible, to contribute to this PRIME-MRM resource as part of PRIME-DE (see Discussion and Data Availability statement).

## Methods

All of the animal procedures performed were approved by the local Animal Welfare and Ethical Review Body and UK Home Office, and the research complies with the Animal Scientific Procedures Act (1986) on the care and use of animals in research and with the European Directive on the protection of animals used in research (2010/63/EU). All persons involved in this project were licenced by the U.K. Home Office and the work was strictly regulated by both institutional controls and the UK Home Office. This publication has been prepared following the Animal Research Reporting of In Vivo Experiments (ARRIVE) principles on reporting animal research.

Subjects are four male rhesus macaques (*Macaca mulatta*) with ages ranging between 10 and 12 years, and weighing between 11 and 14 kg. Subjects were pair housed with another partner in pens ranging from 130 cm × 240 cm to 215 cm × 240 cm. All pens were 230 cm high, and hatches between neighbouring cages were used to increase the space available to the animals. In all four cases the animals were involved in a neuroscientific research protocol, involving techniques such as electrophysiological and/or functional MRI measurements. Animals were acclimated to these techniques using reinforcement training. Such protocols additionally require the use of cranial implants for precise neuronal recordings from brain areas. MRI-compatible Polyether ether ketone (PEEK) head posts are used, which could contain a cylindrical recording PEEK chamber (Cases 1, 3 and 4). The MRI-compatible gentamicin infused cement implant used ceramic screws to anchor the head post, both of which are implanted under aseptic conditions during general anaesthesia and with comprehensive post-operative care. The incidence of adverse effects is low, with very few animals being affected from a colony typically consisting of over 40 animals at any given point in time and over 200 in the last 20 years (also see broader UK data and incidences reported in (Pickard et al., 2013)).

For this particular study, many of the scans were performed with the monkeys awake and behaving during their studies. Thus, routine scanning in parallel to study progression was possible and could help to identify abnormalities before clinical signs are apparent. One scan was performed with the animal anaesthetised (Fig. 7F). Both types of scans (awake and anaesthetised) used similar parameters. The anaesthetised scan has been performed together with the veterinary team using a routine anaesthetic regimen: ketamine (10 mg/kg intramuscular (i.m.)), followed by propofol to effect for induction and maintenance with inspired sevoflurane (~2%) and oxygen.

The MRI scans were taken with a nonhuman primate dedicated, vertical 4.7 Tesla research MRI scanner (Bruker BioSpin, Ettlingen, Germany). 4 channel receive surface coils (Windmiller Kolster Sci., see Table 1 for more details) and a custom transmit coil were used for the awake animal scans. A one channel Bruker birdcage transmit and receive coil was used for the anesthetised scans. We used a range of scans: T1 weighted Magnetization Prepared Rapid Acquisition Gradient Echo (MPRAGE), T2 weighted Rapid Acquisition with Relaxation Enhancement (RARE), Proton Density (Fast low angle shot, FLASH) and time of flight angiography (FLASH ANGIO).

**Table 1:** Diagnostic sequences used and recommended. A basic set of scans includes T1 and T2 anatomical scans, supplemented with diffusion-weighted, proton density and time of flight angiography scans as needed. TR: repetition time; TE: echo time; TI: inversion time; Resolution: 0.6 mm isotropic; FOV (field of view): 10.7 cm × 10.7 cm × 5 cm.

	TR	TE	Other parameters
T1 scan: MPRAGE	2000 ms	750 ms	TI = 750 ms
T2 scan: RARE	14,000 ms	56 ms	
Proton density (PD): FLASH	14 ms	4 ms	Flip angle = 10°
Time of flight angiography:FLASH ANGIO	30 ms	5.1 ms	Flip angle = 60°
Diffusion weighted (Dw) SE-EPI	14,200 ms	58 ms	60 diffusion directions,*b* = 850 s/mm^2^

The processed scans were converted into Analyze format to be loaded into a Medical Image Data Examiner (AMIDE) or FSL View (Smith et al., 2004; Woolrich et al., 2009; Jenkinson et al., 2012) and the angiography scans were pre-processed, segmented and converted to a surface mesh in ImageJ (NIH). All the surfaces displayed have been smoothed and rendered in the image processing and 3D rendering software Blender.

In addition to the MRI monitoring, we perform bi-annual X-rays. These X-rays allow us to monitor bone/implant integration or osteolysis around the screws holding the implant. Some of these x-ray scans were useful as part of the case monitoring (Figure 7E), where information on bone integrity is needed and cannot be unequivocally obtained with ‘black bone’ techniques using MRI (Eley et al., 2017).

MRI quantification of affected areas and comparisons to PRIME-DE normative cohort

*Case 1:* the suspected area of supra-dural abscess was semi-manually segmented for volumetric measurement (in mm^3^) using the *ImageJ* software to seed and segment the affected area. Signal in the suspected abscess region was also compared to signal in a broad ROI of the healthy brain (white and grey matter), which was conducted in the same animal and with PRIME-DE as normative data after normalizing the scanner signal. Fig. 2 shows the quantification results and the variability in the typical brain signal from the PRIME-DE cohort as a reference (10 healthy male macaques drawn from the Newcastle PRIME-DE cohort).

Case 2: In the T2-w images, signal was measured in two ROIs placed respectively in the optic chiasm and a control region (substantia nigra). The substantia nigra was chosen as a reference because of its relative proximity to the optic chiasm and as an area not expected to be affected by a demyelinating condition affecting vision. The ratio of signals in both regions was calculated for Case 2 in a span of 4 years and compared to the 10 healthy male macaques from the Newcastle PRIME-DE cohort as a reference (see Fig. 4).

Case 3: The affected area was separately segmented for volumetric measurement in T1-w and T2-w images using the same semi-manual thresholding approach as in Case 1. The volume of the affected area (in mm^3^) is shown in Fig. 6 separately for both T1 and T2 images. As a reference, the normative data from PRIME-DE do not show such affected areas, thereby a volume of 0 mm^3^ is the point of reference in Fig. 6.

Case 4: ROIs were defined in the suspected abscess and oedema areas, when present, and compared to an unaffected brain control region as in Case 1 or to the PRIME-DE cohort. Reported are signal and standard deviation values as a ratio of the affected areas, relatives to key procedures and time course of resolution observed with MRI (see Fig. 8).

## Results

Case 1: MRI identified suspected infection underneath implant, behaviourally asymptomatic

**Summary:** The first case was behaviourally asymptomatic. MRI monitoring during research scanning led to incidental identification and treatment of an infected area underneath the implant. Treatment effect was monitored with MRI and the infection fully resolved without any notable behavioural impact on the animal.

**History:** Monkey 1 (Case 1) was 12 years old, weighing 13 kg. Case 1 contributed to a study that resulted in several behavioural (Wilson et al., 2013,Wilson et al., 2015a; Milne et al., 2018), EEG (Attaheri et al., 2015), electrophysiological (Kikuchi et al., 2017; Kikuchi et al., 2019) and MRI (Wilson et al., 2015b) reports. Case 1 had an MRI-compatible PEEK head post implanted with ceramic screws and gentamicin cement. The implantations of the post and chamber and the subsequent removal of the chamber were uneventful. Eating and drinking and social interaction behaviours remained normal, and no behaviours associated with pain or discomfort (e.g., head holding or rubbing) were displayed at any time.

**Fig. 1:**
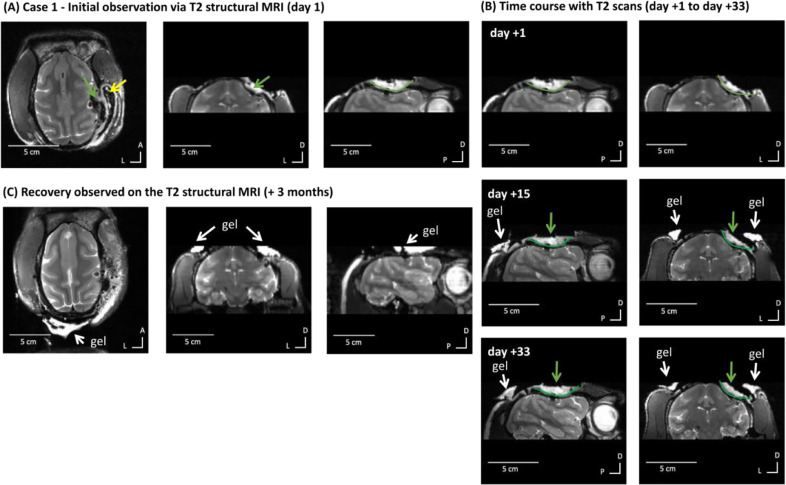
Case 1 - MRI identifies and monitors treatment response of suspected infection. (A) Initial observation with T2 structural MRI (green arrow shows area of interest of affected MRI signal). MRI signal drop out underneath yellow arrow is not clinically significant, this is an expected and unremarkable loss of MRI signal around the chamber which is not visible on MRI scans. From left to right: axial, coronal and sagittal views. (B) T2 structural MRI showing reduction in affected area after treatment (green arrows and lines) from day +1 to day +33. (C) Recovery of affected area observed on T2 structural MRI (+3 months). The white regions around the head are caused by a hydrous gel (identified with white arrows) used to improve the overall scanning quality and thus are not clinically of interest.

**Fig. 2:**
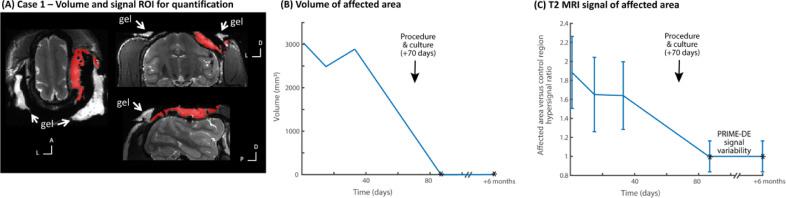
Case 1 – MRI quantification of effects and resolution. (A) shows in red the region of interest (ROI) defined for the affected area (not clinically significant signal from the aqueous gel used to improve imaging quality is identified with white arrows). (B) shows the volume of the area (in mm^3^). Procedure and culture at +70 days is identified and was followed by resolution with no evidence of the suspected abscess (expected volume of 0 mm^3^ affected area based on normative data from PRIME-DE monkeys without evidence of abscess in MRI scans, shown by * in the plot). (C) shows the T2 MRI signal hyperintensity of the affected area relative to the unaffected brain area ROI as a control and the variability of the signal in the affected area (standard deviation); * shows the return to normal signal in the brain area, ratio value = 1, relative to signal variability from the PRIME-DE normative data cohort.

M1 was clinically normal and well. A routine anatomical scan showed a fluid filled area above the dura causing compression over the dorsal sensory motor cortex (Fig. 1A). The finding prompted a review of historical scans. The previous MRI scan had been conducted four months previously and the area could just about be seen on that scan, so it was concluded that the condition had been progressing relatively slowly. Following discussions with veterinarians and human neurosurgeons a most likely diagnosis of extra-axial infection was made, and antibiotic treatment was started (Clavulanate-potentiated amoxicillin, 12.5 mg/kg twice a day (BID) given orally (p.o.) and then Enrofloxacin, 10 mg/kg i.m.). MRI monitoring of treatment response showed minimal response to the treatment over 2–4 weeks (Fig. 1B). Fig. 2 shows MRI quantification data of the affected volume (Fig. 2B) and the T2 signal in the affected area relative to a control region (unaffected white and grey mater) in the same animal or in normative data in 10 macaques drawn from PRIME-DE (see Methods).

Next, an aseptic procedure under general anaesthesia was carried out in order to sample and remove the potential source of infection and to assist diagnosis, treatment and resolution. Material removed during surgery confirmed the presence of a bacterial infection. During the MRI guided procedure the overlying cement was removed and the area cleaned and resealed. Antibiotic treatment based on the culture sample was carried out for 13 days post-procedure with confirmation of resolution (Fig. 1C; Fig. 2B). Case 1 remained clinically unaffected and is completing the final components of the research study.  

Case 2: Eyesight deterioration, suspected naturally occurring demyelinating condition

**Summary:** The second case was a spontaneously occurring suspected degenerative condition of an animal with apparent visual deficit, which could not be attributed to ocular pathology or any procedures. The MRI scans showed demyelination of the optical tracts. The experimental plan for the animal was modified to remove visual tasks and progress to completion of the experimental plan.

**History:** Monkey 2 (Case 2) was 12 years old, weighing around 14 kg. Case 2 was involved in a study that resulted in behavioural (Wilson et al., 2013) and MRI (Wilson et al., 2015b) publications involving auditory tasks. He had an MRI-compatible PEEK head post implant anchored with ceramic screws and gentamicin cement. His initial implant failed and the overlying skin area was closed, after which he contributed to the development of a non-invasive facemask and helmet head immobilisation system (Slater et al., 2016), until re-implantation for the neurophysiological components of the study allowed placing an implant with a recording chamber stereotaxically over the right hemisphere. Case 2′s neurophysiology component involved optogenetics (adeno-associated virus, light sensitive neuronal channel construct) injection into auditory cortex in the temporal lobe, which is well removed from the affected site in the optic chiasm and is unlikely to have led to this degenerative condition; the first indication of optic chiasm signal change (Fig. 4B, time -2 years) preceded the start of the optogenetics injections as part of the study. The implant and skin edge remained stable.

Gradually Case 2 started to show signs of visual impairment without discomfort (decreased hand-eye coordination), he was still able to perform his visual task and was otherwise well, so the decision was to monitor him for signs of deterioration. To assess visual sight deterioration he was sedated for a full ophthalmic exam. The results of the ophthalmic exam were unremarkable with no signs of ocular abnormalities or lenticular opacity for cataract. Subsequently an awake retinoscopy procedure was performed by an ophthalmologist in order to determine the basic refractive state: long-sighted (hyperopia), short-sighted (myopia) and/or astigmatic (not-spherical). Case 2 had retinal reflex equally bright in both eyes and no significant medial distortion in either eye, thus the cornea and lens appeared normal in each eye. There was no significant refractive error in either eye. We could not exclude a low degree of myopia or hypermetropia, but unlikely to be more than +/−1.00D. Both eyes appeared to have a spherical refraction with minimal astigmatism (<0.50D). Intraocular pressure measurements with the iCare tonometer was R20 L21 (within normal limits for an adult human), which greatly reduced the likelihood of glaucoma being responsible for the visual deficit. The visual deficit was not related to an experimental procedure either and therefore was a spontaneously occurring abnormality. Case 2 was prescribed glasses to wear during the behavioural training in order to determine whether it would improve his testing performance. At no point did Case 2 object to wearing the glasses, which were set up for him to engage with voluntarily, or seem in any way adversely affected by using them. However his visual testing performance was also not improved by the use of the glasses.

**Fig. 3:**
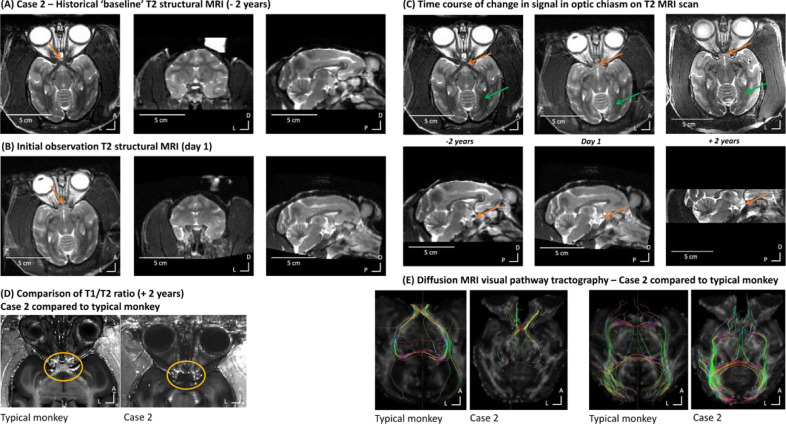
Case 2 – MRI identification of naturally occurring demyelinating condition. (A) Initial historical baseline T2 structural MRI during unaffected vision (2 years prior). No obvious sign of demyelination around the optic chiasm is seen – white matter around optic chiasm (orange arrow) looks typical. From left to right: axial, coronal and sagittal views. The white column in the coronal image is sterile solution used to visualize the location of the recording chamber. (B) Initial observation of T2 structural MRI two years after the historical scan (day 1) where weaker T2 signal in the optic chiasm is seen. (C) Evolution over time of the visual pathway with T2 structural MRI scans (axial and sagittal views). The orange arrow suggests demyelination of the optic chiasm, the green arrow shows less affected myelination of the rest of the optic tract. (D) Comparison of the optical chiasm from a T1/T2 ratio image (myelin weighted) in Case 2 (right) and a typical monkey (left). The orange circle shows the area around the optic chiasm. (E) Diffusion MRI tractography comparison between Case 2 and a typical monkey in the optical tracks from the optic chiasm (left two images) or focusing on the optical tracts starting from visual cortex (right two images).

After an anatomical MRI scan and in comparison with an older historical scan (Fig. 3A-B), we observed a change in the composition of the optical pathway myelination. The optical chiasm appeared darker in the historical T2-weighted scan as expected (Fig. 3A) but less dark in the more recent ones (Fig. 3B). This contrast change suggested demyelination of the optical tract from the eyeball to the optic chiasma (Fig. 3C). The rest of the brain and other pathways seemed to be visually unchanged.

**Fig. 4:**
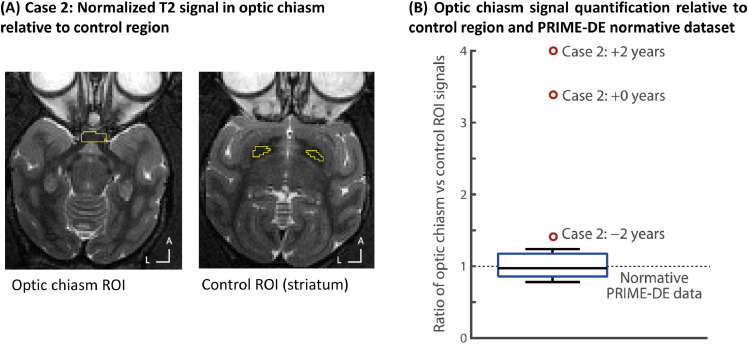
Case 2 – MRI quantification of effects and progression. (A) Regions used for quantification of the MRI T2 signal in the optic chiasm and a control region in the striatum (yellow outline regions). (B) shows the ratio of the optic chiasm relative to the control ROI T2 signal; the box-plot shows the normative distribution from the PRIME-DE cohort. Note how Case 2 at first identification (-2 years) is already well outside of the normative distribution and there is progression of signal loss (higher control vs. optic chiasm ratio values) at 0 and +2 years.

We also compared his scans with other animals (Figs. 3D; 4). The comparison showed an affected MRI signal on Case 2′s T2 structural scan (orange circles in Fig. 3D, right panel) suggesting a change in the composition of the optic chiasm, which would be consistent with demyelination. The comparison of the visual pathway tractography using diffusion weighted MRI and tractography analyses also showed white matter tractography differences around the optic chiasm. The first tractography analysis (Fig. 3E) focused on the visual pathway starting from the optic nerve to the primary visual cortex (V1) (Fig. 3E, left panels) and the second tractography analysis from V1 toward the optic nerve (Fig. 3E, right panels). These two comparisons highlight the different pathways the optical tracts take with Case 2 compared to a typical animal, particularly around the optic chiasm.

Fig. 3E shows that the fibres coming from V1 follow the same cortico-thalamic path all the way to the optic chiasm for Case 2 and the ‘healthy animal’ but in Case 2 do not seem to cross at the optic chiasm as they should. Both comparisons (Fig. 3D-E) support a diagnosis of optic chiasm demyelination as the cause of the visual impairment. Fig. 4 shows quantitative MRI data comparing T2 signal in the optic chiasm to a nearby control region (striatum) and a reference comparison in normative data from the PRIME-DE cohort; note the progressive change in signal in the optic chiasm in this Case (Fig. 4B).

There was no evidence on the MRI scans of tumour or pressure on the area, and glaucoma had been previously ruled out because the intraocular pressure measurements were normal. Neurology consultation suggested a ‘chiasmal syndrome’ which may be associated with an immune inflammatory response with unknown cause (Foroozan, 2003) or possibly multiple sclerosis (MS). Indeed, it has been shown in humans that one of the initial manifestations of MS is an inflammatory demyelinating disorder of the optic chiasm (Foroozan, 2003; Lee et al., 2012; Milea and LeHoang, 2002; de Champfleur et al., 2013).

Case 2's visual impairment continued to very gradually deteriorate until he was unable to discriminate small objects from background when presented in front of him (i.e., nuts, raisins) but could move around his home environment without problems as long as the environment and the location of objects was familiar. It is not clear if steroid treatment would have been able to slow down or reverse the deterioration in visual ability and the impact on the optic chiasm. We considered it as part of this animal's treatment in consultation with the neurology team and consultants, but decided to forego steroid treatment because it could have affected the integrity of the immune system and in human patients there can be spontaneous recovery of visual function. It was decided with the veterinary team and in accordance with Home Office guidance that the experimental plan for Case 2 would be modified to complete the auditory study he was involved in with a terminal procedure, as he was not considered suitable to proceed with any visual tasks. In the meantime, and to improve his comfort, his home unit layout, apparatus, and enrichment remained the same in order to facilitate him being familiar with the environment. This allowed him to move around confidently and without injury. He remained well and otherwise unaffected until completion of his experiment.  

Case 3: MRI signals and time course of resolution of suspected internal cerebral haemorrhage (ICH)

**Fig. 5:**
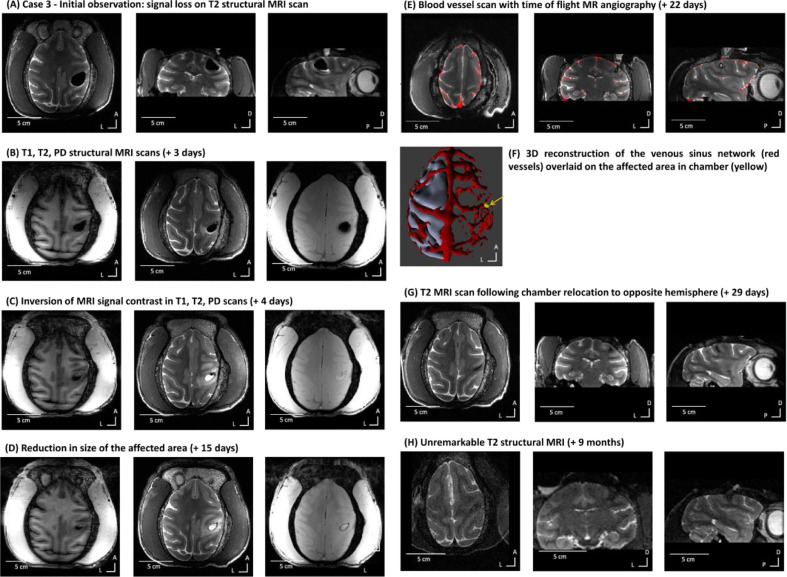
Case 3 – MRI signals and time course of resolution of suspected ICH. (A) Initial MRI identification consisting of MRI signal loss in a T2 structural MRI scan (day 1). From left to right: axial, coronal and sagittal views. (B) Initial T1 and T2 MRI scans of uncertain diagnosis (+ 3 days). From left to right: T1, T2 and PD MRI scans. (C) Inversion of contrast on T1, T2 and PD scans (+ 4 days). (D) Continued reduction in size of the affected area (+ 15 days) on T1, T2 and PD scans. (E) Blood vessel enhancement scan (time of flight MR angiography) overlaid on a T2 RARE structural scan (+ 22 days). (F) 3D reconstruction of the venous sinus network and an overlay of the craniectomy area (yellow). (G) T2 structural MRI after chamber was relocated to the opposite hemisphere (+ 29 days). (H) Unremarkable T2 structural MRI (+ 9 months).

**Fig. 6:**
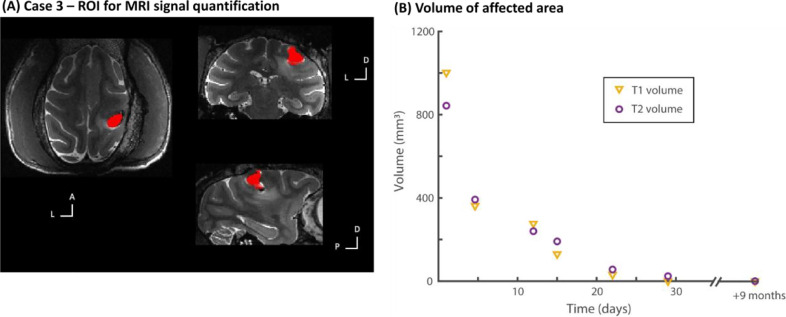
Case 3 – MRI quantification of effects and resolution. (A) shows in red ROI used for quantification. (B) Shows the volume of the affected area for both the T1 and T2 signals. Note how there is substantial reduction and resolution in the affected area after about one month, with return to normal brain signal. Normative PRIME-DE cohort brain scans show 0 mm^3^ of affected areas.

**Summary:** The third case presented with mild left-handed weakness following a minor surgical procedure to create a small craniectomy within a recording chamber. MRI scans showed a suspected intra-axial cerebral haemorrhage in the acute/subacute stage, presenting as an area of MRI signal loss. Time of flight MRI angiography identified an unusually sited subdural venous drainage network directly in the location of the chamber, which was thought to have been accidentally disrupted during the procedure. Continued MRI monitoring confirmed a substantial reduction and resolution in the size of the affected area over 3 weeks combined with MRI signal returning underneath which normal tissue could once again be observed (Fig. 5, Fig. 6). These MRI results were associated with a complete resolution of the left-handed weakness. Future recordings were planned to avoid the location of the subdural venous drainage network, until he completed his experiment.

**History and treatment:** Monkey 3 (Case 3) was 12 years old, weighing around 11 kg. Case 3 was involved in a study which resulted in behavioural, fMRI and electrophysiological publications (Schneider et al., 2018). He had an initial MRI-compatible PEEK head post implant. The initial implant was lost and skin closed after which he worked on a project to develop a non-invasive facemask and helmet head immobilisation system (Slater et al., 2016) until he was recommended for re-implantation and addition of a right hemisphere chamber. The implant and skin edge were stable after re-implantation and x-rays showed good implant integration with the skull. He progressed towards neurophysiological recordings to complete the study that he was on.

Two days after a procedurally unremarkable minor craniectomy inside the chamber (around 5 mm diameter), Case 3 started to show behavioural signs of discomfort such as lethargy, head and eye rubbing and contralateral to chamber hand weakness. Following discussion with the veterinary team, he was recommended for MRI scanning. The initial T1 and T2 contrast MRI-monitoring scans showed an area of MRI signal loss (~2 cm diameter), involving the hand motor cortex region (Fig. 5A), surrounded by a layer of oedema seen on the T2 scan. The absence of MRI signal on any of the contrasts (hypointense on both T1 and T2) and a small amount of watery fluid at the bottom edge of the affected area (hyperintense signal in Fig. 5A) identified the area affected. Consultation with MRI physicists, veterinarians and neurosurgeons included consideration of possible air through a breach in dura and/or ICH because of an affected subdural vessel.

Initial treatment involved anti-inflammatories (meloxicam, 0.2 mg/kg p.o. and dexamethasone, 0.5 mg/kg i.m. initially and then a decreasing dose across 5 days) and MRI-monitoring. MRI monitoring combined with daily veterinarian checks to assess demeanour, behaviour and neurological function showed a substantial reduction of the MRI signal loss in this area (around 20.0 mm diameter at +3 days, ⁓12.0 mm at +7 days; Figure 5B-C) and a resolution of the T1 and T2 signal in the area (Figure 5D, + 15 days), along with a resolution of the clinical signs. Diagnosis remained uncertain and did not follow the reported ICH resolution on T1 and T2 signal as in human cases (Bradley Jr, 1993), suggesting that there may have been a small amount of air causing MRI signal loss with oedematous fluid. As his clinical signs were improving he remained under close monitoring for a further two weeks.

After the course of the two weeks of monitoring, MRI showed a change of contrast with opposite T1/T2 contrasts in two areas. This was seen as an external T2 hyperintense signal with T1 hypointense signal (suspected fluid) and an internal T2 hypointense signal with T1 hyperintense signal, indicative of potential subacute ICH occurring albeit with a delay of 3 days after the surgical procedure, thus not directly associated with the procedure itself (Fig. 5C).

Subsequently, the T2 scans showed an inversion of contrast of the internal mass, consistent with resolution of a minor slow ICH as expected from human MRI involving ICH events (Bradley Jr, 1993). MRI monitoring showed there to be a continued steady decrease in overall size (Fig. 5D; involving T1 and T2 scans with more periodic PD scans). Fig. 6 shows quantification of the T1 and T2 signals and the time course of resolution; note that the PRIME-DE reference point is 0 mm^3^ volume of such unaffected areas.

As these scans appeared to support the possibility of ICH and a small air pocket, it was decided to obtain an angiogram scan (Fig. 5E-F). Angiography identified a venous sinus network just underneath the chamber thought to have resulted in a slow ICH in the days after surgery (Fig. 5E-F). After 29 days from first observation, the affected area of MRI signal had reduced substantially (Fig. 5G). Four months after the event, the chamber was relocated to the opposite hemisphere in order to avoid the venous sinus seen on the 3D MRI angiography scan (Fig. 5G). Although the diagnosis remains tentative relating to ICH due to the delayed scale of T1 and T2 resolution compared to that expected in human clinical patients with ICH (Bradley Jr, 1993), the management of the case was greatly facilitated by the information gained from MRI monitoring and the outcome was successful.

After seven months without any sign of discomfort or abnormal behaviour or weakness, Case 3 showed a slight tremor of the left hand, opposite to the location of the previously affected area. This did not affect his ambulation, feeding, foraging or social behaviour in the colony. A week afterwards, Case 3 had a hemi-epileptiform episode (affecting the left side of the body) that was immediately treated with and responded to midazolam (0.25 mg/kg i.m). In the post-dromal phase there was mild weakness in left limbs which returned to normal after 2 hours. With MRI monitoring, the T2 MRI scan was unremarkable compared to the previous one (Fig. 5H; also see Fig. 6B). Because the cause of the epileptiform activity was unclear, to avoid any risk of further epileptiform activity in discussion with the veterinary team and Home Office inspectors it was decided that the animal should be euthanised to ensure conclusion in a controlled and elective way.  

Case 4: MRI identification, treatment and resolution of suspected abscess

**Summary:** The fourth case presented with mild left-handed weakness contralateral to a recording chamber over sensory motor cortex above the central sulcus. MRI imaging identified two suspected subdural abscess sites. These were targeted for antibiotic treatment and either aspiration or implant modification. MRI monitoring also facilitated the tracking of antibiotic treatment response and abscess resolution. Thereafter the animal's hand use returned to normal.

**History and treatment:** Monkey 4 (Case 4) was a 10 year old monkey, weighing around 11 kg. Case 4 had contributed to behavioural and MRI work (Rinne et al., 2017; Wikman et al., 2019) prior to neurophysiological work in preparation. He had a prior MRI-compatible PEEK head post implant and a recording chamber was positioned stereotaxically over the right hemisphere to target superior temporal cortex. The implant and skin edge had been stable.

**Fig. 7:**
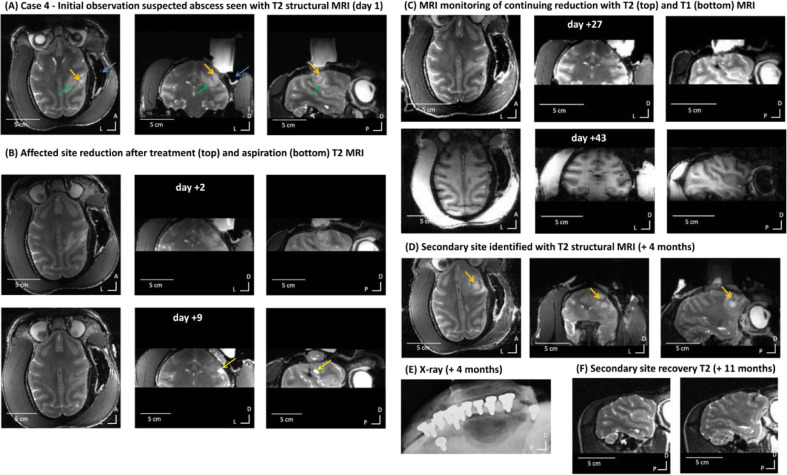
Case 4 - MRI identification, treatment and resolution of suspected abscess. (A) Initial observation with T2 structural MRI (day 1). Orange arrow over the affected area centre and green arrow shows the oedema penumbra. Blue arrow points to MRI signal dropout that is not clinically significant (expected lack of MRI signal from the cement around the chamber that is not visible on MRI scans). Scans shown from left to right: axial, coronal and sagittal views. (B-C) Affected site resolution time-course following treatment and monitoring with T2 structural MRI. (D) Secondary site identification and monitoring with T2 structural MRI (orange arrows). (E) X-ray monitoring of implant integration; x-ray image shows where the cement implant above the skull can be seen as well as the ceramic anchoring screws. Shadowing around screws may indicate bone retraction. This x-ray shows that the implant is intact and well integrated apart from two screws with shadowing (in the front and middle), which was also confirmed visually during the implant removal procedure. (F) Secondary affected site resolution after implant removal confirmed with T2 MRI scans.

Following a superficial chamber infection, Case 4 began showing mild lethargy, then head and eye rubbing that responded to pain medication (meloxicam 0.2 mg/kg) and a temporary intermittent left-hand weakness with some twitching involving his left lip, cheek and eyebrow. The initial T2 MRI monitoring scan identified an area (⁓5 mm diameter, orange arrows in Fig. 7A) surrounded by affected signal (suspected oedema, green arrows in Fig. 7A), which was seen as hyper-intense signal on the T2 scan. A tentative diagnosis of subdural abscess located under the recording chamber was made.

After consultation with all parties, further MRI monitoring was recommended in conjunction with antibiotics (ceftiofur, 2.0 mg/kg subcutaneous (s.c.), changed to clavulanate-potentiated amoxicillin, 12.5 mg/kg BID p.o. once culture and sensitivity results were received), anti-inflammatories (meloxicam, 0.2 mg/kg p.o., dexamethasone, 0.5 mg/kg i.m. initially and then reducing the dose over 5 days) and additional analgesics (paracetamol, 10 mg/kg p.o.).

**Fig. 8:**
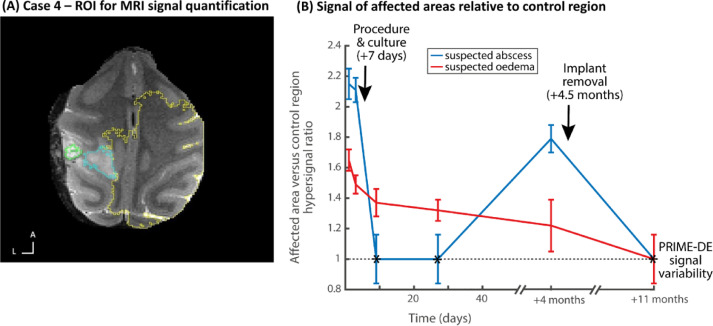
Case 4 – MRI quantification of effects and resolution. (A) shows on an axial MRI image the regions of interest (ROI) defined for the affected areas (suspected abscess in green outline, surrounding oedema in blue outline) and a reference region in the rest of the brain (yellow outline). (B) shows the T2 MRI signal hyperintensity of the affected areas relative to the unaffected brain area as a control and the variability of the signal in the affected area (standard deviation); * shows the return to normal signal in the brain area, ratio value = 1, relative to signal variability from the PRIME-DE normative data cohort.

Subsequent MRI monitoring showed a substantial reduction in affected area size (Fig. 7B). Fig. 8 shows quantification of the suspected abscess and oedema sites in relation to a control brain area in the animal or in relation to expected normative signal from the PRIME-DE cohort. At this point a subdural MRI-guided procedure to aspirate the centre of the suspected abscess was recommended by neurology consultants. This procedure was performed seven days after the beginning of treatment and after application of antibiotics, after which the craniectomy was sealed with a silicon plug to prevent contamination (Fig. 7B; Fig. 8B). Following this procedure the abscess site further greatly reduced and all clinical signs resolved with the oedema site more gradually (Fig. 8B). Subsequently, the animal continued to be clinically normal with no return of clinical signs or MRI abnormalities (Fig. 7C).

Four months later, a secondary site was incidentally observed on MRI-monitoring T2 scan distant to the chamber location (Fig. 7D; see quantification in Fig. 8B). This coincided with Case 4 showing signs of discomfort such as rubbing of the head and hunched back sitting position, although no left hand weakness. This was promptly treated with and responded to meloxicam (0.2 mg/kg p.o.). The suspected abscess was treated with antibiotics (enrofloxacin, 10 mg/kg i.m.).

After 10 days of treatment and evidence of response to treatment, in consultation with the UK Home Office inspectors it was agreed that this site could not be treated in the same way with an aspiration procedure and that, assuming the source of infection was the cranial implant, that the best way to treat it would be to remove the implant. During the surgery to remove the implant, mild osteomyelitis was observed around some of the screws (also seen as shadowing with X-rays taken at the same time, see Fig. 7E). The rest of the implant and screws were firmly integrated. The area above the affected site, however, showed a localized infection that had not been visible with the implant in place.

Implant removal was successful, the skin completely closed over the area of the implant and the animal was treated post operatively with antibiotics and non-steroidal anti-inflammatory medication. MRI scans confirmed resolution of the abscess (Fig. 7F). This case has been behaviourally and with MRI-monitoring scans unremarkable to date with no further treatment necessary and no clinical signs.

This case also underscores the usefulness of combined MRI and X-ray monitoring for diagnosing and monitoring treatment response. The animal was transiently mildly to moderately affected during the period of treatment, but he has since been normal with no clinical signs and unremarkable MRI scans. Most recently he has been working on a touch screen behavioural task as part of his experimental plan completion.

## Discussion

MRI monitoring has informed diagnosis, treatment and allowed visualization of treatment response, as we consider in detail further below in relation to each of the cases. Complications can arise even in neurological and neurosurgical work with human patients, thereby MRI assessment and monitoring is an important clinical tool that is available in many hospitals. Because of refinement in neuroscientific and neurosurgical procedures, the incidence of adverse effects in primates is low (e.g., see broader UK data and incidences reported in (Pickard et al., 2013)). Thereby, a primate MRI monitoring resource and a community-wide effort are needed to achieve sample sizes typically not possible to obtain at any one given institution. We provide information on how to access and contribute to the resource further below.

This study demonstrates the potential usefulness of MRI monitoring with macaques taking part in neuroscientific work important for biomedical scientific advances. The study also underscores the importance of collaboration and consultation across researcher, veterinary, neurology and regulatory teams, in order to reach a plan for the most appropriate treatment and monitoring. We also establish a *PRIMatE MRI Monitoring* (PRIME-MRM) resource as part of the PRIME-DE primate open data sharing initiative (Milham et al., 2018, 2020). In so doing, we both contribute data to and rely on data from PRIME-DE to quantitatively compare the cases to normative data in PRIME-DE. Quantitative MRI data from a given human neurology case is often compared to other patients of the same type and to normative data, informing patient-specific diagnosis, treatment and decision-making, whenever the patient's results are well outside of normative distributions. While this approach is available for human patients, outside of MRI information from specific disease models (e.g., Parkinson's disease (Luan et al., 2008), stroke (Li et al., 2018)), MRI monitoring more broadly for nonhuman primates on neuroscientific procedures is largely missing, and a resource and framework for sharing these type of data are needed, as noted by (Bridge et al., 2019; Prescott and Poirier, 2020; Basso et al., 2021). However, if established such an approach and resource could be of substantial benefit for animal welfare and in support of biomedical and scientific advance.

Case 1 highlights the importance of MRI monitoring because the monkey was behaviourally asymptomatic. Early identification of an unexpected area of infection under the implant was treated and resolved. If MRI monitoring had not been possible, the affected area would have been more substantial and likely to have led to intra-cranial pressure and oedema. This would only have been picked up after indication of a behavioural impact on the animal at a much later stage, thereby impairing wellbeing. As we show, Case 1 MRI monitoring not only helped in early identification but also in showing that the initial treatment response was not as effective as it could have been (Fig. 2B-C). This was immediately followed by a procedure to culture and procedurally clean the affected area, which led to confirmation of diagnosis, improvement of treatment, treatment monitoring and resolution and the animal continuing not to show any obvious behavioural signs.

Case 2 had visual problems, the cause of which could not be identified with ophthalmic inspection nor corrected with glasses. MRI showed demyelination around the optic chiasm far removed from the implant, suggestive of a multiple sclerosis type condition or other autoimmune disorder. The animal apart from the visual difficulties was otherwise behaviourally unremarkable and well. However, MRI monitoring helped narrow the potential diagnosis possibilities (Fig. 4), as would also be the case with such information in a human patient. This type of information informed decision making based on the specific impact on the optic chiasm, ruling out other forms of peripheral and central visual impairment. Continuing degeneration (Fig. 4B) also informed decision making to amend and complete the scientific work with the animal. A different type of visual cortex-dependent impairment has previously been reported in a macaque case (Bridge et al., 2019).

With Case 3 MRI monitoring identified an unexpected complication and although a definitive diagnosis was not possible, MRI was again essential for narrowing down the diagnostic possibilities and for monitoring response to treatment. The approach also shows how angiography can be useful in research planning to avoid venous networks and we outline a set of basic and extended imaging scans that can be used for MRI monitoring (Table 1). Unfortunately although the animal stabilized and the MRI monitoring led to a decision to move the recordings to the opposite hemisphere, which were uneventful, a hemi-epileptic event contralateral to the previously affected area combined with unremarkable MRI scans led to the decision that the humane endpoint for the animal was reached, as a safety precaution to prevent the small potential risk of further episodes.

With Case 4 we showed how MRI monitoring assisted in the treatment of two sites of suspected abscess, where the treatment approach for both was very different. MRI showed the time course and confirmed resolution of both (Fig. 8). The animal was stabilised after treatment and is doing well. In both Cases 3 and 4 there were only relatively subtle changes in behaviour associated with their condition. A case has previously been reported of MRI guided removal of silicon foreign body in a monkey with a plastic recording chamber, which, alongside the current study, highlights the importance of MRI guided-treatment (Doane et al., 2018).

In all of these cases, MRI monitoring was extremely useful. Without this information, detection of their conditions would have been delayed, diagnosis less certain and treatment potentially less effective at a much more progressed stage. Also treatment monitoring would have been much less precise without the MRI information on the affected brain areas.

This paper establishes a *PRIMatE MRI Monitoring* (PRIME-MRM) resource as part of PRIME-DE (https://fcon_1000.projects.nitrc.org/indi/indiPRIME.html). The scans contributed to this PRIME-MRM resource are available for further analysis and we anticipate will grow with contributions from the scientific community that can achieve larger sample sizes. To access or contribute, search for the PRIME-MRM label in the main PRIME-DE repository or in the individual animal ‘dataset_description’ and ‘participant’ .json and .tsv file information. For general guidance on metadata contributions, see: Poirier et al. (2021). Key parameters to include are diagnostic information and date of event(s), if known, with links to the scanning data contributed in PRIME-DE. Updated information on how to access and contribute to the resource can be found at the PRIMatE Research Exchange (PRIME-RE: https://prime-re.github.io/; (Messinger et al., 2020)) under the MRM resource page: https://prime-re.github.io/hardware_and_protocols/prime-mrm.html.

This paper also shows an approach for quantifying effects in relation to normative data in PRIME-DE. To minimise the influence of inherent signal fluctuations in the scan data between animals, scanners, sequences etc., our approach relied on within dataset controls or signal normalised quantification when comparing to other scans or datasets. Such quantitative information was very useful and is preferred over qualitative impressions that rely on more subjective and less precise visual comparisons of scans across different time points.

For these cases we consulted with veterinary and neurology teams to assist in the diagnosis and treatment plan. As part of this resource, we also establish a Clinical And Radiology Expert (PRIME-CARE) consultation network (contact information is available on the PRIME-MRM resource page: https://prime-re.github.io/hardware_and_protocols/prime-mrm.html).

We encourage the primate neuroscientific community to grow this type of information, given the general availability of MRI scanners at universities and the increasing number of locations throughout the world able to scan monkeys regularly with MRI (Milham et al., 2018, 2020). These sites could share information on at least periodic MRI monitoring, provided that there are no welfare or MRI contra-indications (Milham et al., 2018, 2020), as we consider below. This will help to grow the evidence base on the aspects of MRI monitoring that are most beneficial alongside the types of scans that are useful for managing certain cases, in either awake or anesthetised scanning states. Practical procedural information is also being shared to assist the community (Messinger et al., 2020; Basso et al., 2021; Poirier et al., 2021).

We recognise that MRI monitoring is not always feasible, advisable or available. Thereby each site needs to consider the practical and animal welfare costs alongside the approach that might be most useful for MRI monitoring. As examples, metal implants are MRI contraindicated, an MRI scanner may be unavailable or scanning could be too expensive to use on a regular basis. MRI incompatible implants would preclude MRI monitoring in the animals. However, even in animals with MRI incompatible implants, many sites conduct pre-implant planning with MRI. Those data can be shared, particularly if an incidental case is found and treated (e.g., our Case 1). There are also now many sites capable of anaesthetised and awake MRI monitoring with MRI compatible implants that can also grow this resource (Milham et al., 2018, 2020). Moreover, there are ongoing refinements of chronic implants, including MRI compatible implants (Johnston et al., 2016; Ortiz-Rios et al., 2018). While the MRI-compatible implants used in these cases can be stable for years in the vast majority of the cases, customised, MRI-compatible hydroxyapatite coated implants are being developed and tested (Ortiz-Rios et al., 2018). Developments such as these could be assessed and compared to other implant options using MRI monitoring (Prescott and Poirier, 2020).

MRI monitoring cannot be prescribed in every case, because the benefits of awake or anesthetised MRI scanning, even if possible at a given site, need to be weighed in relation to the cost to the animal's welfare, if that animal is judged not to be able to tolerate the scanning. It is important to note that because work at Newcastle University involves regular functional MRI in awake behaving animals—to assist in translating scientific insights to humans by way of neuroimaging as a bridging technique to humans—our animals are trained for several months to acclimate to and to work in the scanner. As such our research groups specialise in awake macaque scanning (Milham et al., 2018), which requires regular MRI scanner training, and because of this allows for more frequent monitoring while avoiding general anaesthesia effects. This may not be possible in many places. Community-wide refinements in primate anaesthesia protocols have improved (Murphy et al., 2012; Basso et al., 2021). However, there might still be a risk of anaesthesia in a given animal, depending on the case, which may not justify the potential benefit of conducting MRI monitoring, and decisions should be made at the level of the individual. This dearth of information however could be offset by sites that are able to conduct more regular MRI monitoring and provide more detailed MRI monitoring information for certain cases. For instance, the cases presented here contain a substantial number of MRI scans at different time points, providing important information for research and veterinary teams when planning the ideal time to scan and treat (or the duration of treatment). This information for similar cases might amount to deciding to conduct a single scan at a crucial point in time to identify an affected area that can be acted upon, potentially with a later scan to visualise the treatment response, if possible. Or the second scan can be skipped by relying on the anticipated evidence from the first MRI scan of behavioural improvement, which we've seen as a good indication of MRI resolution. If MRI monitoring is possible, however frequent or infrequent, we recommend obtaining both a T2 and T1 weighted structural scans (taking ⁓15 min to acquire), complemented with other more event specific scans as needed and time permits (e.g., Proton Density, Angiography time of flight).

Finally, our scanner being a vertical 4.7 Tesla is different from the more commonly available 3 and 1.5 Tesla horizontal scanners that are used to scan nonhuman primates. Although there are advantages in scanning with higher resolution at higher magnetic fields, we did not push the resolution of the scans and the effects seen can also be well visualised and quantified at lower resolution. Moreover, 4.7T and 3T scanner quality is generally comparable across the scanning sites as part of PRIME-DE (Milham et al., 2018). Lastly, none of the effects we noted were so small that they required a high-resolution and higher-field scanner to visualise.

Infrequent X-rays allow assessing bone integrity and, as we show in Case 4, can also be useful, but would need to be assessed with more data and/or in relation to computed tomography (CT) scanning. For sites that have such scanners, CT might be preferred for assessing large internal bleeds or can be useful for evaluating infection when combined with leukocyte labelling (Guerriero et al., 2019). However we like some other institutions, did not have one available for use in order to obtain CT data to compare to MRI. This would be another welcome addition to the PRIME-MRM resource. A further important facet for future work, is the use of contrast agents, which help to enhance signal from affected areas and assist in patient diagnosis. None of the cases reported here required contrast agent (e.g., gadolinium), as effects seen were robust and culture and sensitivity analysis from tissue sampling confirmed the diagnosis of infection whilst providing important information regarding potential treatments (as would have also been possible had contrast agent been used). Because of the unknown potential impact of gadolinium deposition in brain and other tissues (Guo et al., 2018), we reserve the use of contrast agents for cases where there is a definite need. However, where scanning results are ambiguous, contrast agent use could be a useful addition to the scan protocols described here.

In summary, we establish a PRIME-MRM nonhuman primate MRI monitoring resource, which could be further assessed as the resource grows as part of PRIME-DE. This could lead to the early identification of potential issues, and assist in localisation of affected sites, diagnosis and monitoring of the treatment response. While this is shown here with cases from a relatively large primate species, the same approach could be useful with smaller primates (Sadoun et al., 2015) and other animal species. However, this aspect would need to be assessed and established in those species separately with the appropriate scanners, parameters and sequences and contributed to the PRIME-MRM and PRIME-DE resources neither of which are intended to be species specific.

## Declaration of Competing Interest

None.
